# Discovering a Multi-Component Combination against Vascular Dementia from Danshen-Honghua Herbal Pair by Spectrum-Effect Relationship Analysis

**DOI:** 10.3390/ph15091073

**Published:** 2022-08-29

**Authors:** Peilin Zhang, Shiru He, Siqi Wu, Yi Li, Huiying Wang, Changyang Yan, Hua Yang, Ping Li

**Affiliations:** State Key Laboratory of Natural Medicines, China Pharmaceutical University, No. 24 Tongjia Lane, Nanjing 210009, China

**Keywords:** Danshen-Honghua herbal pair, spectrum-effect relationship, multi-component combination, vascular dementia

## Abstract

The Danshen-Honghua (DH) herbal pair exhibits a synergistic effect in protecting the cerebrovascular system from ischemia/reperfusion injury, but the therapeutic effect on vascular dementia (VaD) has not been clarified, and the main active ingredient group has not been clarified. In this work, the chemical constituents in DH herbal pair extract were characterized by UHPLC-QTOF MS, and a total of 72 compounds were identified. Moreover, the DH herbal pair alleviated phenylhydrazine (PHZ)-induced thrombosis and improved bisphenol F (BPF)- and ponatinib-induced brain injury in zebrafish. Furthermore, the spectrum-effect relationship between the fingerprint of the DH herbal pair and the antithrombotic and neuroprotective efficacy was analyzed, and 11 chemical components were screened out as the multi-component combination (MCC) against VaD. Among them, the two compounds with the highest content were salvianolic acid B (17.31 ± 0.20 mg/g) and hydroxysafflor yellow A (15.85 ± 0.19 mg/g). Finally, we combined these 11 candidate compounds as the MCC and found that it could improve thrombosis and neuronal injury in three zebrafish models and rat bilateral common carotid artery occlusion (BCCAO) model, which had similar efficacy compared to the DH herbal pair. This study provides research ideas for the treatment of VaD and the clinical application of the DH herbal pair.

## 1. Introduction

Vascular dementia (VaD), the major type of dementia except for Alzheimer’s disease (AD), is a neurocognitive impairment syndrome associated with cerebrovascular injury [1]. In the progression of VaD, intense inflammatory responses and excessive reactive oxygen species (ROS) are accompanied by a cerebrovascular blockade, which triggers endothelial cell dysfunction and neuronal apoptosis, resulting in damage to cognition and memory [2,3,4]. VaD has a high mortality rate [5]. However, there is no specific drug for it [6]. Several FDA-approved anti-AD drugs, such as acetylcholinesterase inhibitors and memantine, show modest efficacy against VaD [7]. Therefore, the discovery of effective drugs to improve and treat VaD is an important problem that needs to be solved urgently.

Chinese medicine herbal pairs involve the application of two Chinese medicines, which are the relatively fixed minimum prescription units in traditional Chinese medicine compound recipes (TCMCRs) and have special significance in clinical practice. This can not only improve the efficacy but also reduce toxicity and side effects. Salviae Miltiorrhizae Radix et Rhizoma (Danshen, DS), the dried root and rhizome of *Salvia miltiorrhiza* Bunge., is widely used in the treatment of coronary heart disease, angina pectoris, myocardial infarction, and other diseases. Carthami Flos (Honghua, HH) is the dried flower of *Carthamus tinctorius* L., which is applied in the treatment of cardiac-cerebral vascular diseases, gynecological diseases, and traumatic injuries. Importantly, DS and HH are commonly used as one herbal pair for blood-activating [8]. The Danhong injection (DHI) is made from the aqueous extracts of DS and HH at a dose ratio of 3:1. It is often used in the clinical treatment of coronary heart disease, angina pectoris, and ischemic stroke [9,10]. Studies suggest that the Danshen-Honghua (DH) herbal pair has a potentially beneficial effect on VaD. It has been reported that the main pharmacologically active components of the DH herbal pair are polyphenolic acids, diterpene compounds, carthamin, and hydroxysafflor yellow A [11]. Since the therapeutic effects of most herbal pairs are based on the synergistic effects of their multi-components and multi-targets, it is not sufficient to elucidate the role of only one or two markers or bioactive ingredients in complex preparations. In addition, the content of bioactive compounds is closely related to genotypes, climatic conditions, plant phenology, and other factors, which makes the compatibility of traditional Chinese medicines (TCMs) more complicated. Therefore, it is urgent to establish a specific and sensitive screening method for the principal components in TCMs to assure their efficacy, safety, and quality.

The pathological mechanisms of VaD are complex, and the rat bilateral common carotid artery occlusion (BCCAO) model is often used to simulate VaD in pharmacological experiments. However, large-scale drug screening cannot be carried out due to the disadvantages of complex operations, high cost, and poor reproducibility. Zebrafish are regarded as an important model organism for drug screening due to the advantages of high throughput and low cost. Moreover, with the high genome homology and similarities in brain anatomy and physiology to humans, zebrafish have received increasing attention in neurological research [12,13]. Considering that thrombosis is an important factor leading to cerebral infarction and the formation of VaD, phenylhydrazine (PHZ) can induce acute thrombosis with local blood stasis by causing oxidative damage to the lipid membrane of erythrocytes and increasing the rate of thrombin production [14]. Furthermore, reduced cerebral blood flow leads to an insufficient nutritional supply for neurons, ultimately resulting in impaired neuronal function and apoptosis during the pathological process of VaD [15]. Bisphenol F (BPF), a neurotoxic agent that induces apoptosis in the central nervous system, was used to establish the neuronal injury model of zebrafish [16]. Moreover, a larval zebrafish ischemic stroke model induced by ponatinib was developed and validated under an optimized exposure concentration and treatment period in our laboratory. The pathophysiology of this ischemic stroke animal model demonstrated high similarities with human ischemic stroke and could be used for disease study and drug screening [17].

In our study, the antithrombotic and neuroprotective effects of the DH herbal pair were confirmed using the zebrafish blood stasis model and zebrafish neuronal injury model. Next, by connecting it with chemical information, a potential bioactive multi-component combination (MCC) was obtained through a spectrum-effect relationship analysis, and the improvement effects of the MCC were equivalent to the DH herbal pair. Moreover, the MCC effectively alleviated the spatial memory impairment of VaD rats, inhibited ROS eruption, and protected hippocampus neurons from apoptosis, which had similar efficacy compared to the DH herbal pair. These results indicated that the MCC was the active ingredient group of the DH herbal pair against VaD. This study offers recommendations for further research into the treatment of VaD and the clinical use of the DH herbal pair. It also provides a reference for the study of the complex components of TCM.

## 2. Results

### 2.1. Characterization of the Chemical Constituents in the DH Herbal Pair

The chemical constituents of the DH herbal pair extract (DS: HH 1:1, g/g) were characterized by ultra-high-performance liquid chromatography coupled with quadrupole time-of-flight tandem mass spectrometry (UHPLC-QTOF MS). The representative total ion chromatograms (TIC) in both positive and negative ion modes are presented in Figure S1. A total of 72 compounds were identified, including 20 phenolic acids, 14 diterpenoid quinones, 20 flavonoids, 9 organic acids, 3 amino acids, 2 terpenoids, 1 purine, 1 phenylpropanoid, 1 furfural, and 1 polyacetylene. Among them, 24 compounds were unambiguously identified with reference standards, and the others were tentatively characterized with the relative literature according to the retention time, product ions, and fragmentation pathways. Their detailed MS data are shown in Table 1.

### 2.2. The DH Herbal Pair Alleviated PHZ-Induced Thrombosis in Zebrafish

**Figure 1:**
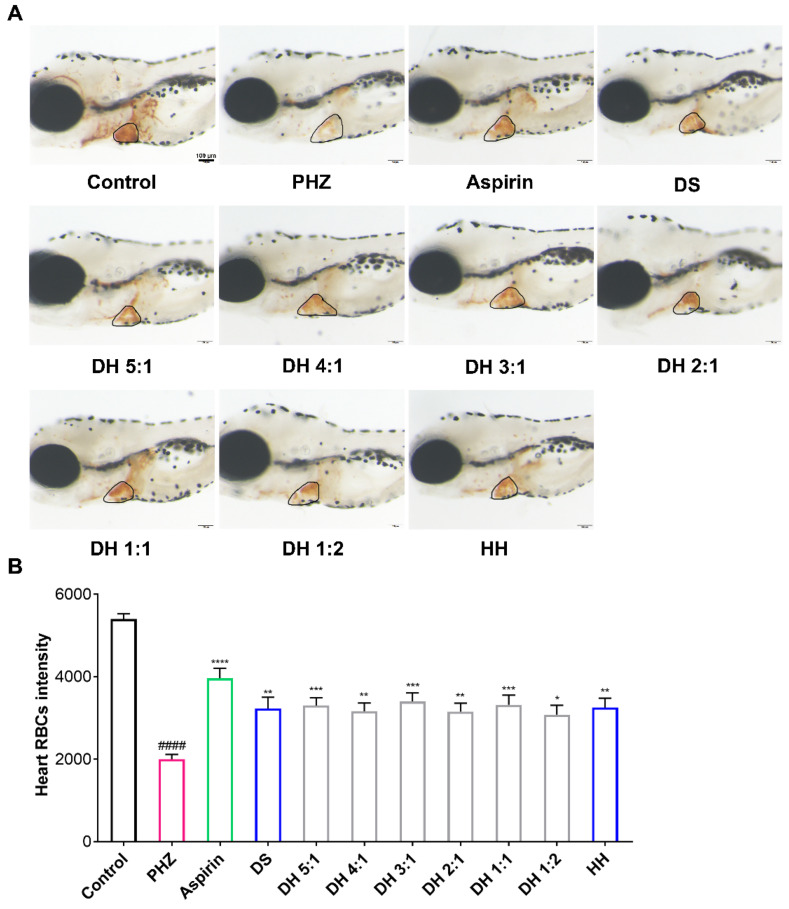
Danshen-Honghua (DH) herbal pair alleviated phenylhydrazine (PHZ)-induced thrombosis in zebrafish. The zebrafish thrombosis was induced by PHZ (1.5 μM) for 24 h, and the antithrombotic efficacy of different proportions of the DH herbal pair was evaluated with the heart red blood cell (RBC) intensity. (**A**) Staining of heart RBCs in different groups. (**B**) The heart RBC intensity (in the black circle) was quantitatively analyzed with Image Pro Plus 6.0 software. The data are presented as mean ± SEM (n = 10). DS: Danshen; HH: Honghua; DH: Danshen-Honghua. * *p* < 0.05, ** *p* < 0.01, *** *p* < 0.001, **** *p* < 0.0001 vs. PHZ group; #### *p* < 0.0001 vs. control group.

Before the experiment, we investigated the maximum nonlethal concentration (MNLC) of different compatible proportions of the DH herbal pair in zebrafish, and the results showed that 50 μg/mL was the MNLC; therefore, we chose 50 μg/mL as the dose of the DH herbal pair (Table S1). As shown in Figure 1, PHZ significantly reduced the heart red blood cell (RBC) intensity in zebrafish compared with the control group, indicating successful thrombosis modeling. Aspirin, as a positive drug, had an improving effect on this model. After being cultured with DS or HH, the intensity of RBCs in the heart position of zebrafish significantly increased, and the DH herbal pair, with different compatible proportions, also showed similar efficacy, suggesting that DS, HH, and the DH herbal pair had significant antithrombotic effects. The thrombosis inhibition rates are shown in Table 2.

### 2.3. The DH Herbal Pair Improved Brain Injury of Zebrafish

**Figure 2:**
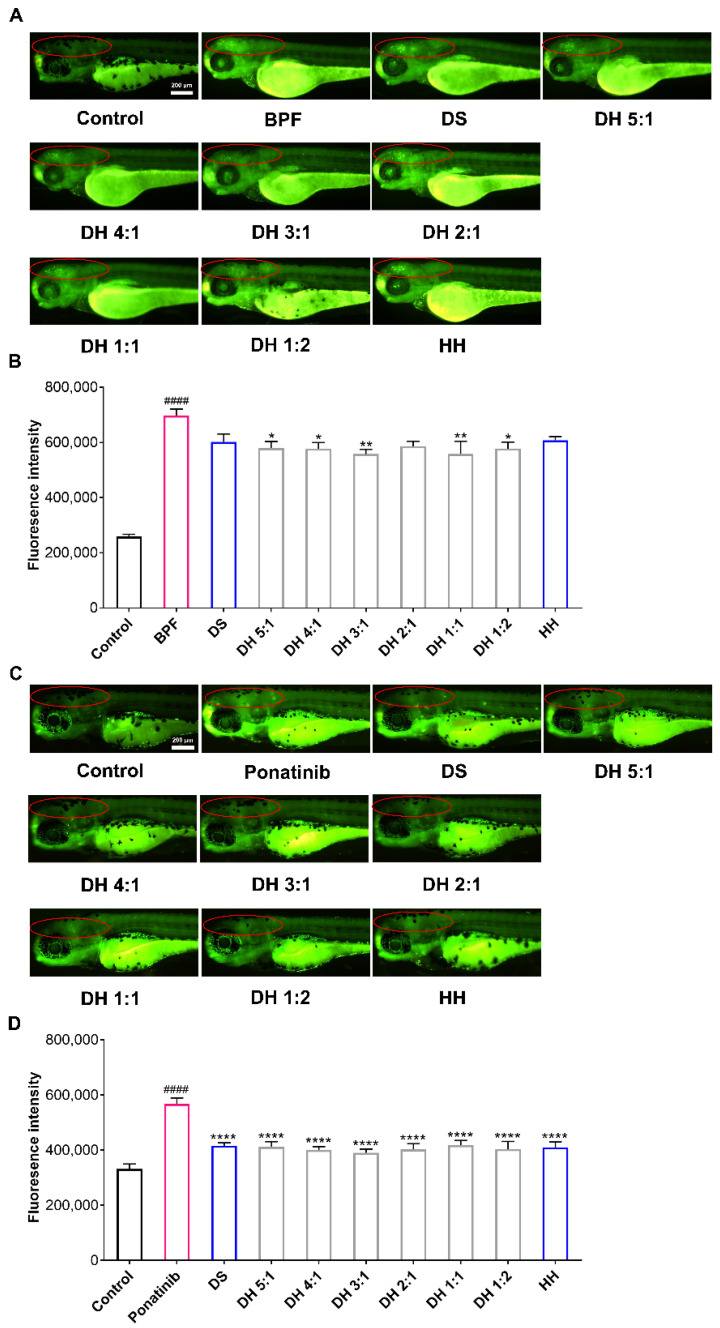
The DH herbal pair improved the brain injuryof zebrafish. (**A**) The zebrafish neurological injury model induced by bisphenol F (BPF) (10 µg/mL) and the neuroprotective efficacy of DS, HH, and the DH herbal pair. (**B**) After treatment with BPF or designated drugs, the fluorescence intensity in the brains (in the red circle) of the zebrafish was quantified with the Image J software. (**C**) The zebrafish ischemic stroke model induced by ponatinib (1 μg/mL) and the neuroprotective efficacy of DS, HH, and the DH herbal pair. (**D**) After treatment with ponatinib or designated drugs, the fluorescence intensity in the brains (in the red circle) of the zebrafish was quantified with the Image J software. The data are presented as mean ± SEM (n = 10). DS: Danshen; HH: Honghua; DH: Danshen-Honghua. * *p* < 0.05, ** *p* < 0.01, **** *p* < 0.0001 vs. model group; #### *p* < 0.0001 vs. control group.

For the BPF-induced neuronal injury in zebrafish, the MNLC was determined at 75 μg/mL (Table S2). As shown in Figure 2A,B, BPF stimulation significantly enhanced fluorescence intensity in the brains of the zebrafish compared with the control group, which indicated that neuronal apoptosis occurred in the brains of the zebrafish. Compared with the model group, the DH herbal pair treatment contributed to reducing the fluorescence intensity of the zebrafish brains, in which the combinations of 5:1, 4:1, 3:1, 1:1, and 1:2 of the DH herbal pair had a significant improvement effect on neuronal injury (Table 2).

Similarly, we determined 75 μg/mL to be the dose of the DH herbal pair by conducting the MNLC investigation (Table S3). As the results show in Figure 2C,D, after a 24 h administration of 1 μg/mL of ponatinib, the fluorescence intensity of the zebrafish brains in the model group was significantly increased compared with the control group, indicating neuronal injury and cerebral ischemia occurred in zebrafish. DS, HH, and the DH herbal pair treatments all reversed the increase in fluorescence intensity in the brain, suggesting a single drug or the herbal pair of the two drugs exerted a significant neuroprotective role (Table 2).

### 2.4. Discovering Potential Bioactive Components of the DH Herbal Pair Based on Spectrum-Effect Relationship Analysis

**Figure 3:**
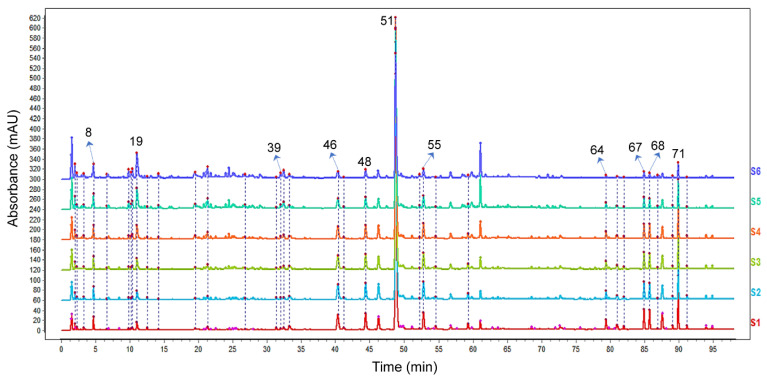
HPLC fingerprint of six proportions of the DH herbal pair. The chromatograms of S1–S6 represent the following: DH 5:1 (S1); DH 4:1 (S2); DH 3:1 (S3); DH 2:1 (S4); DH 1:1 (S5); DH 1:2 (S6).

**Figure 4:**
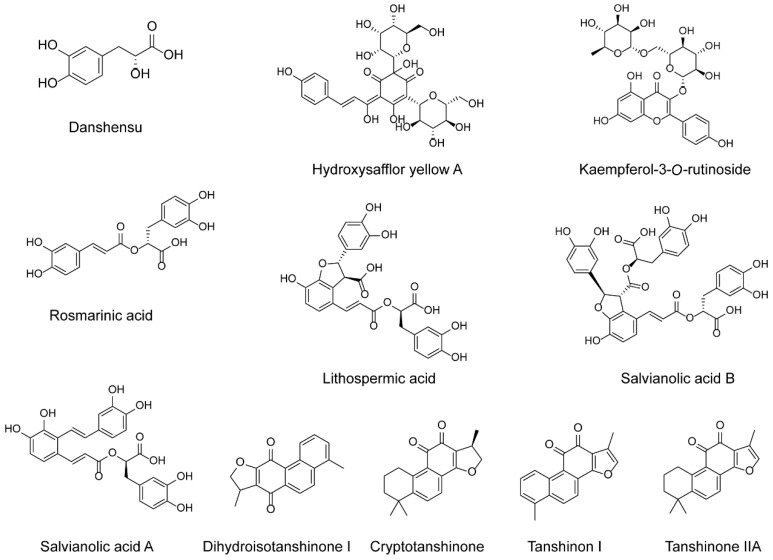
Chemical structures of 11 potential active components in the DH herbal pair.

In our study, the partial least squares (PLS) model was used for the spectrum-effect relationship analysis, which was built by relating the fingerprint of the DH herbal pair to the efficacy of the zebrafish models. Taking a VIP value > 1 as the screening criterion, peaks 8, 19, 39, 46, 48, 51, 55, 64, 67, 68, and 71 were closely related to the efficacy of the DH herbal pair in three zebrafish models (Table 3), implying these 11 components were potential bioactive ingredients in the DH herbal pair against VaD. With their MS data, as well as by authenticating with reference standards, the 11 compounds were unambiguously identified as danshensu, hydroxysafflor yellow A, kaempferol-3-*O*-rutinoside, rosmarinic acid, lithospermic acid, salvianolic acid B, salvianolic acid A, dihydrotanshinone I, cryptotanshinone, tanshinone I, and tanshinone IIA, respectively. As shown in Figure 3, the positions of 11 compounds in the HPLC characteristic chromatogram were labeled, and the chemical structures are shown in Figure 4.

### 2.5. Determination and Preparation of a Multi-Component Combination

Next, we combined these 11 candidate compounds as the MCC to compare their efficacy against VaD with that of the DH herbal pair. It is necessary to prepare the MCC according to the content proportion of the original recipe. Therefore, a quantitative analysis of the chemical composition of the DH herbal pair extract with a 3:1 compatibility ratio was carried out with an Agilent 1290 liquid chromatography system. As listed in Table 4, the two compounds with the highest content were salvianolic acid B (17.31 ± 0.20 mg/g) and hydroxysafflor yellow A (15.85 ± 0.19 mg/g). The content of the other compounds ranged from 0.89–3.03 mg/g.

All the calibration curves showed excellent linearity with correlation coefficients (r^2^) of more than 0.9994. The limits of detection (LODs) and quantification (LOQs) were 0.000–0.195 μg/mL and 0.007–0.977 μg/mL, respectively. The relative standard deviations (RSDs) of repeatability and inter- and intraday precision were 1.11–3.32%, 0.20–1.69%, and 0.50–2.81%, respectively, indicating good precision and repeatability. The 11 analytes were stable in the prepared sample solution at 4 °C within 24 h, with RSDs of 0.47–1.41%. Recoveries of the 11 analytes ranged from 95.76% to 101.80% with RSDs between 1.13% and 4.04%, which showed the method had good accuracy (Table S4).

### 2.6. The MCC of the DH Herbal Pair Improved Thrombosis and Neuronal Damage in Zebrafish

**Figure 5:**
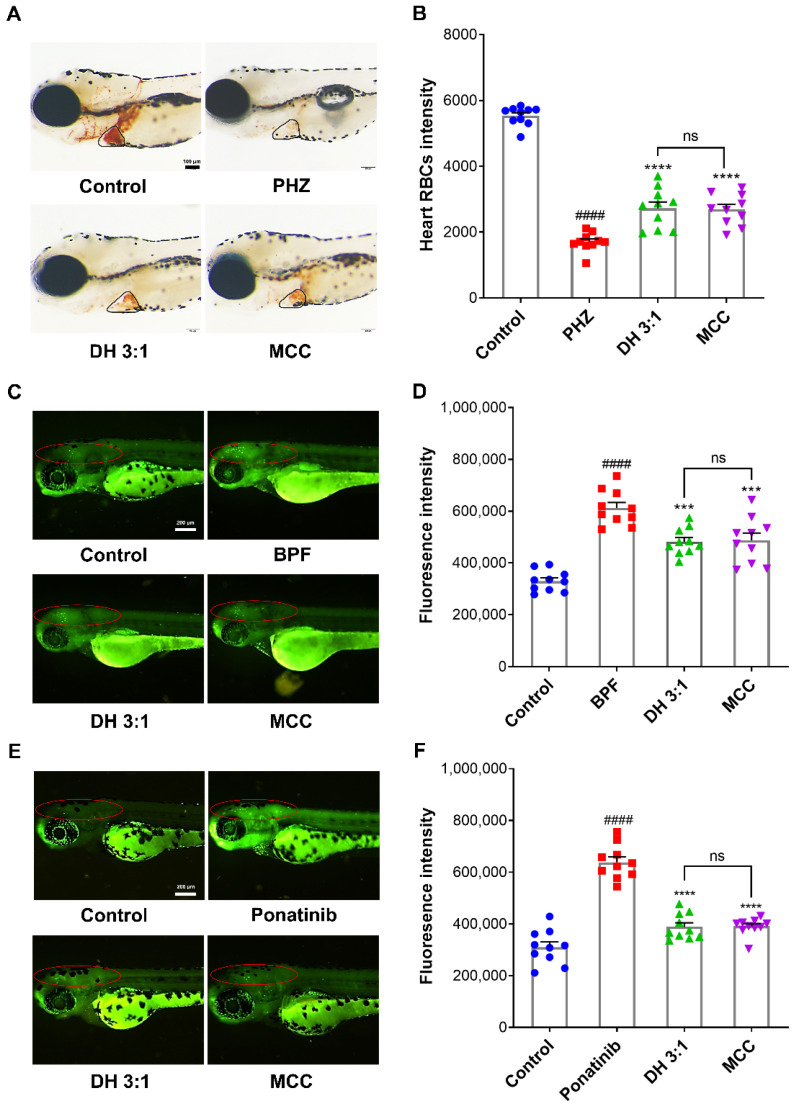
The multi-component combination (MCC) improved thrombosis and neuronal injury in zebrafish. (**A**,**B**) Zebrafish thrombosis was induced by PHZ (1.5 μM), and the antithrombotic efficacy of the DH herbal pair and MCC was evaluated by calculating the heart RBC intensity (in the black circle) with the Image Pro Plus 6.0 software. (**C**,**D**) The zebrafish neurological injury model was induced by BPF (10 µg/mL). (**E**,**F**) The zebrafish ischemic stroke model was induced by ponatinib (1 μg/mL). The neuroprotective efficacy of the DH herbal pair and MCC was analyzed by calculating the fluorescence intensity in the zebrafish brains (in the red circle) with the Image J software. The data are presented as mean ± SEM (n = 10). DH: Danshen-Honghua *** *p* < 0.001, **** *p* < 0.0001 vs. model group; #### *p* < 0.0001 vs. control group; ns: no significant difference.

In the model of PHZ-induced thrombosis in zebrafish, the MCC group significantly increased the intensity of heart RBCs (*p* < 0.001), and the antithrombotic effect was equivalent to that of the 3:1 combination of the DH herbal pair (*p* = 0.9978) (Figure 5A,B). Similarly, in the BPF-induced neurological injury model and ponatinib-induced ischemic stroke in larval zebrafish, treatment with the MCC significantly reduced the green fluorescence intensity of the zebrafish brains and alleviated neuronal injury (*p* < 0.001). The DH herbal pair also had a protective effect on neurons, and there was no significant difference compared with the MCC (*p* = 0.9967 & *p* = 0.9998), suggesting equivalence (Figure 5C–F).

### 2.7. The MCC of the DH Herbal Pair Improved Cognitive Impairment in VaD Rats

**Figure 6:**
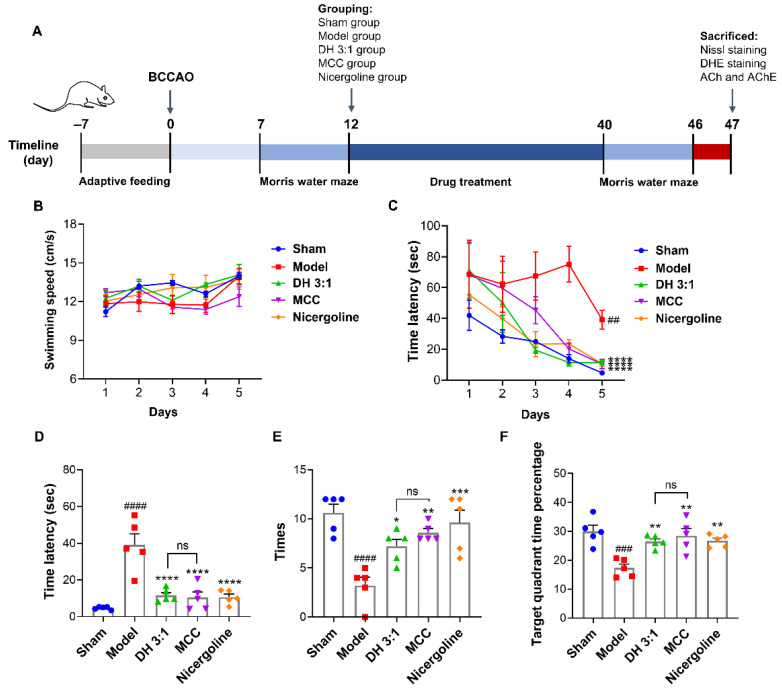
The MCC of the DH herbal pair improved cognitive impairment in VaD rats. (**A**) The time schedule of animal experiment protocols. (**B**,**C**) The swimming speed and the escape latency of rats from day 41 to day 45 in the Morris water maze (MWM) test were recorded. (**D**) The escape latency on day 45 in the MWM. (**E**,**F**) The platform crossing times and the target quadrant time percentage within 120 s were recorded on day 46 after the platform was removed. The data are presented as mean ± SEM (n = 5). DH: Danshen-Honghua; MCC: multi-component combination. * *p* < 0.05, ** *p* < 0.01, *** *p* < 0.001, **** *p* < 0.0001 vs. model group; ## *p* < 0.01, ### *p* < 0.001, #### *p* < 0.0001 vs. sham group; ns: no significant difference.

A Morris water maze (MWM) was carried out to evaluate the learning and spatial memory behavior of rats. Next, we established the rat BCCAO model to further investigate the regulatory effect of the MCC on cognitive impairment in VaD rats (Figure 6A). As shown in Figure 6B, there was no significant difference in swimming speed among all groups, demonstrating that surgery would not cause movement disorder in rats. After five days of acquisition training, the escape latency of all animals declined compared with that on the first day (Figure 6C). Rats in the model group showed longer escape latency than in the sham group after 5-day training (Figure 6C,D). When the platform was removed, the platform crossing times, as well as the target quadrant time percentage, were also reduced in the model group, indicating the learning and spatial memory performance of these rats were severely impaired (Figure 6E,F). Treatment with the DH herbal pair and the MCC markedly decreased the escape latency and increased the platform crossing times and target quadrant time percentage (Figure 6B–F). It was noteworthy that a significant difference was not found in the MCC group and the DH herbal pair group (*p* = 0.9993 & *p* = 0.7908 & *p* = 0.9034), which suggested the MCC was equivalent to the original herbal pair in improving the memory capacity and spatial memory retention of VaD rats. Nicergoline, a commonly used clinal drug for dementia (including AD and VaD), was chosen as a positive control in our study. It had similar effects to the DH herbal pair and also significantly improved cognitive impairment and memory in VaD rats (Figure 6B–F).

### 2.8. The MCC Showed an Equivalent Neuroprotective Effect to the DH Herbal Pair in VaD Rats

**Figure 7:**
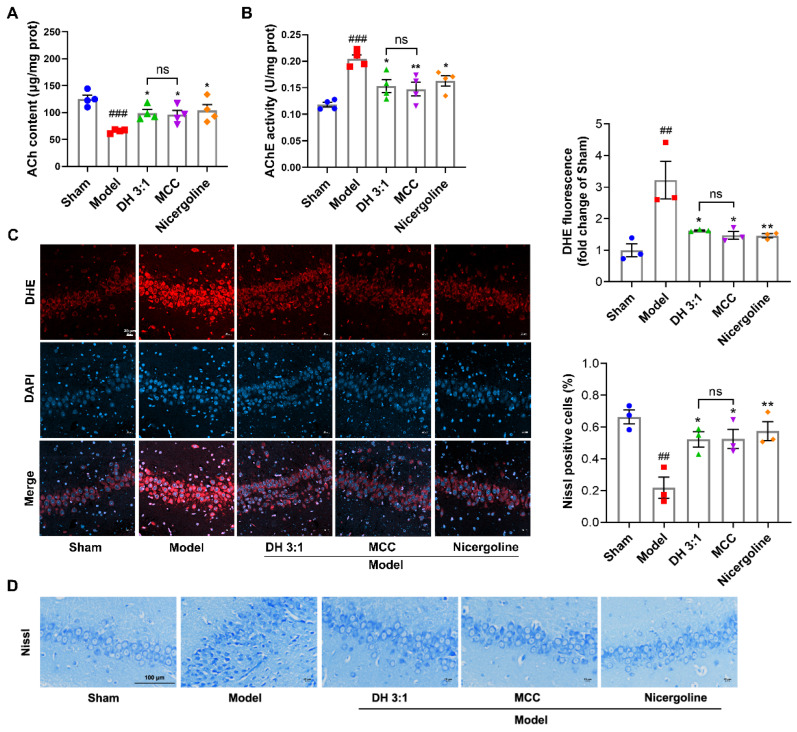
The MCC showed an equivalent neuroprotective effect to the DH herbal pair in VaD rats. (**A**,**B**) Acetylcholine (ACh) content and acetylcholinesterase (AChE) activity in the hippocampal tissues were measured. (**C**) ROS production in the hippocampal CA3 area was labeled using DHE staining (scale bar = 20 μm). (**D**) The neuronal damage in the hippocampal CA3 area was observed using Nissl staining (scale bar = 100 μm). The data are presented as mean ± SEM (n ≥ 3). DH: Danshen-Honghua; MCC: multi-component combination. * *p* < 0.05, ** *p* < 0.01 vs. model group; ## *p* < 0.01, ### *p* < 0.001 vs. sham group; ns: no significant difference.

The central cholinergic system is involved in learning, memory, navigation, cognition, and other processes of organisms. Acetylcholine (ACh) is an important neurotransmitter produced by the cholinergic system, the level of which can be regulated by the specific cholinergic marker acetylcholinesterase (AChE) in cholinergic neurons [18,19]. ACh deficiency in the cerebral cortex and hippocampus has been reported to cause cognitive impairment in AD and VaD patients [20,21]. As expected, a significant decrease in ACh levels and an increase in AchE activity in the hippocampus tissues were observed in model rats compared with those in the sham group (Figure 7A,B). The DH herbal pair partially restored ACh levels and decreased AchE activity, and similarly, the MCC ameliorated the dysregulation of the cholinergic system in a manner indistinguishable from the DH herbal pair (*p* = 0.9880 & *p* = 0.9936) (Figure 7A,B). As a response to ischemic signals, the balance of the redox state within the mitochondria was disrupted, resulting in the eruption of ROS and progressive neuronal damage [15]. To assess the ROS level in the hippocampus, DHE staining was carried out. Compared with the sham group, DHE fluorescence signal intensity in the hippocampal CA3 area was significantly increased in the model group, revealing more ROS accumulation, while it was reversed after the DH herbal pair and MCC treatments (Figure 7C). Moreover, the survival of hippocampal neurons was evaluated with Nissl staining. In the sham group, the neurons in the hippocampal CA3 area were closely arranged and the morphology was clear. On the contrary, hippocampal neurons in the model group were significantly lost and irregular in shape. After the DH herbal pair and MCC treatment, the neurons were densely arranged and restored to maintain a complete structure (Figure 7E). It should be noted that there was no significant difference between the MCC group and the DH herbal pair group (*p* = 0.9954 & *p* > 0.9999), which suggested the MCC was equivalent to the original herbal pair in inhibiting oxidation damage and neuronal injury in VaD rats. These results suggest that the DH herbal pair and the MCC indeed contribute to equivalent effects, improving cognitive function and protecting neurons in VaD rats by improving the cholinergic system and antioxidant activity. A similar beneficial effect was observed with the positive drug nicergoline, further confirming the protective effect of the DH herbal pair on VaD.

## 3. Discussion

There are multi-level and multi-link synergistic relationships between the complex chemical components of TCM, which makes the holistic efficacy of TCM not a simple combination of single components. Bioactive equivalent combinatorial components (BECCs) are defined as a collection of exact compositions that represent the entire efficacy of the original TCM [22,23]. BECCs are helpful in clarifying the pharmacodynamic material basis of TCM and promoting the transformation of Chinese herbal formulae from the traditional form of “unclear” to the modern form of “clear effective components”. In the current study, we screened and obtained the bioactive MCC of the DH herbal pair for improving thrombosis and neuronal injury and further verified that the anti-VaD effect of the MCC was comparable to that of the original herbal pair in three zebrafish models and rat BCCAO model. This study broke through the pharmacodynamic material basis research on the DH herbal pair in the treatment of VaD and promoted the quality evaluation of TCM from “single component, index component, and local evaluation” to “multi-components, main effective components group, and overall evaluation”.

Danhong injection, derived from the DH herbal pair, is a famous traditional Chinese medicine preparation in clinical practice used to improve the outcomes of patients with cardiac-cerebral vascular diseases, such as strokes and angina [24,25]. The DH herbal pair showed a strong blood-activating effect on blood-stasis rats by regulating the parameters involved in hemorheology and the plasma coagulation system [8]. In our study, the DH herbal pair also showed a beneficial therapeutic effect on VaD, which may benefit from its antithrombotic and neuroprotective effects. Due to the complexity of the ingredients in the DH herbal pair, multiple studies have focused on performing the orthogonal compatibility method on the primary effective molecules of this herbal pair. The four effective ingredients in DH (tanshinol, salvianolic acid A, salvianolic acid B, and hydroxysafflor yellow A) have exhibited a protective effect on rats with cerebral ischemia/reperfusion injury, potentially through the inhibition of apoptosis via the downregulation of key targets upstream of the caspase-3 pathway [26]. Moreover, salvianic acid A, protocatechuic aldehyde, salvianolic acid B, and hydroxysafflor yellow A have protective effects on hippocampi hypoxia by inhibiting oxidative stress damage and cell apoptosis, resisting thrombosis, and reducing the intracellular calcium ion of overload [27]. In our study, we screened 11 components of the DH herbal pair as a bioactive MCC; among them, salvianolic acid B and hydroxysafflor yellow A were the two compounds with the highest content. The MCC could improve spatial memory impairment and reverse neuronal injury in the hippocampus of the VaD rats, partly related to the restoration of cholinergic system functions through antioxidant effects.

Zebrafish have become an attractive model animal in recent years due to the characteristics of available embryos, larval transparency, and affordability [28]. Notably, when compared to mammals, the basic structure of the vascular anatomy of zebrafish is conserved, promoting them to become important research objects for cardiac morphogenesis and neurovascular development [29,30]. A PHZ-induced zebrafish thrombosis model could be used for in vivo thrombosis studies and for rapid screening and efficacy assessments of antithrombotic drugs. Six human antithrombotic drugs (aspirin, clopidogrel, diltiazem hydrochloride injection, xuanshuantong injection, salvianolate injection, and astragalus injection) have shown significant preventive and therapeutic effects on thrombosis in PHZ-induced zebrafish [14]. BPF could induce significant neurotoxicity in zebrafish embryos by inducing neuroinflammation and central nervous system (CNS) cell apoptosis [16]. Ponatinib-induced zebrafish have shown thrombosis, reduced blood flow, inflammation, and apoptosis, as well as reduced motility, which is similar to that of human ischemic stroke patients [17]. These zebrafish models could be used to study the complex cellular and molecular pathogenesis of VaD and to rapidly identify therapeutic agents. In our study, we established three zebrafish models and found that different proportions of DH herbal pairs could inhibit thrombosis and prevent neurons from stimulant-induced apoptosis in zebrafish, which achieved high throughput and rapid and reliable efficacy confirmation.

Nowadays, the spectrum-effect relationship analysis plays an indisputable role in elucidating the material basis of TCM, optimizing drug compatibility, improving preparation processes, and, eventually, the development and clinical application of new TCMs [31]. In this study, we combined the fingerprint of the DH herbal pair with efficacy based on zebrafish models and confirmed 11 fingerprint peaks that are closely related to antithrombotic and neuroprotective effects using a spectrum-effect relationship analysis. In addition, it has been reported that the main active components of the DH herbal pair against blood stasis are caffeic acid, salvianolic acid B, hydroxysafflor yellow A, and lithospermic acid [8]. However, caffeic acid was not included in our results, which might be due to different experimental animals, modeling methods, and detection indicators.

In conclusion, we combined the compounds of the DH herbal pair with the efficiency of three zebrafish models and identified a candidate MCC using a spectrum-effect relationship analysis. The candidate MCC was prepared based on the quantitative analysis results of 11 chemical components, which exerted significant antithrombotic and neuroprotective effects on three zebrafish models and rat BCCAO model. This work answers the key question of what the real bioactive components against VaD in the DH herbal pair are and simultaneously provides a feasible method for screening the main active components of the herbal pairs.

## 4. Materials and Methods

### 4.1. Materials and Reagents

Ponatinib was bought from MedChemExpress (St. Monmouth Junction, NJ, USA). PHZ was obtained from Sinopharm Chemical Reagent Co., Ltd. (Shanghai, China). Acetylsalicylic acid (aspirin) was supplied by Aladdin Company (Shanghai, China). Nicergoline was bought from Shandong Qidu Pharmaceutical Co., Ltd. (Shandong, China). Danshensu, protocatechuic acid, neochlorogenic acid, protocatechualdehyde, *p*-hydroxybenzoic acid, chlorogenic acid, cryptochlorogenic acid, caffeic acid, and rutin were purchased from Chengdu Manst Biotechnology Co., Ltd. (Chengdu, China). Hydroxysafflor yellow A, *p*-coumaric acid, kaempferol 3-*O*-sophoroside, quercetin-7-*O*-glucoside, kaempferol 3-*O*-rutinoside, rosmarinic acid, lithospermic acid, salvianolic acid B, salvianolic acid A, salvianolic acid C, dihydrotanshinone I, cryptotanshinone, tanshinone I, and tanshinone IIA were obtained from Chengdu Push Biotechnology Co., Ltd. (Chengdu, China). Anhydrosafflor yellow B and BPF were bought from Shanghai Yuanye Biotechnology Co., Ltd. (Shanghai, China). The purity of all reference standards was higher than 98%. Dimethyl sulfoxide (DMSO) and acridine orange were supplied by Sigma-Aldrich (St. Louis, MO, USA). *O*-dianisidine was bought from J&K Scientific Ltd. (Beijing, China). Formic acid (purity 99%) was obtained from ROE Scientific Inc. (Newark, New Castle, DE, USA). HPLC-grade acetonitrile was purchased from Merck (Darmstadt, Germany). Deionized water was prepared by passing distilled water through a Milli-Q system (Millipore, Milford, MA, USA). Other reagents and chemicals were analytical-grade.

The radix and rhizoma of *Saliva miltiorrhiza* Bge. (Labiatae) and the flowers of *Carthamus tinctorius* L. (Asteraceae) were both purchased from Jiangsu Yifeng Pharmacy Chain Co., Ltd. (Jiangsu, China) in October 2021. The voucher specimens (No. 210,401 and No. E2106030101) were deposited in the State Key Laboratory of Natural Medicines, China Pharmaceutical University, Nanjing 210009, China.

### 4.2. Preparation of Sample Solutions

Eight different proportions of DH herbal pair extracts (DS: HH 1:0, 5:1, 4:1, 3:1, 2:1, 1:1, 1:2, 0:1, g/g) were prepared. A total of 10 g of mixed powder DS and HH was reflux-extracted with 100 mL 75% ethanol for 3 h. After filtration, the solvent was lyophilized by a freeze-dryer system (DZF-6050, Shanghai Jing Hong Laboratory Instrument Co., Ltd., Shanghai, China) to obtain the dried extract.

To investigate the efficacy of the DH herbal pair in the rat BCCAO model, 600 g of mixed powder DS and HH (DS: HH 3:1, g/g) was extracted following the above procedure. After lyophilization, 178.4 g dried extract was obtained with a yield of 29.73%. All the extracts were stored at −20 °C.

### 4.3. HPLC Analysis

The HPLC analysis of DH herbal pair extract with different proportions (20 mg/mL) was performed on the Agilent 1290 liquid chromatography system (Agilent Technologies, Palo Alto, CA, USA). The Agilent Zorbax Extend C-18 column (150 mm × 3.0 mm, 3.5 μm) was used for separation at a flow rate of 0.4 mL/min. The mobile phase consisted of 0.1% formic acid in water (A) and acetonitrile (B). The detailed elution program was as follows: 5–8% B at 0–6 min, 8–10% B at 6–14 min, 10–15% B at 14–23 min, 15–17% B at 23–38 min, 17–22% B at 38–48 min, 22–26% B at 48–58 min, 26–40% B at 58–68 min, 40–45% B at 68–73 min, 45–60% B at 73–83 min, 60–90% B at 83–93 min, 90–100% B at 93–95 min, and 100% B at 95–98 min. The injection volume was 5 μL, the column temperature was 35 °C, and the UV detection wavelength was 280 nm.

### 4.4. Qualitative Analysis of the DH Herbal Pair

The qualitative analysis of the DH herbal pair extract (DS: HH 1:1, g/g) was carried out on the Agilent 6520 QTOF Mass Spectrometer (Agilent Technologies, California, USA) equipped with an electrospray ionization (ESI) mode and coupled to the Agilent 1290 liquid chromatography system. The HPLC condition was the same as in the above HPLC analysis. The MS parameters were as follows: mass range, *m*/*z* 100–1500; drying gas (N_2_) flow rate, 10.0 L/min; drying gas temperature, 350 °C; nebulizer gas pressure, 40 psig; capillary voltage, 3500 V (negative ion mode) and 4000 V (positive ion mode); fragment, 120 V; collision energy, 15 eV, 20 eV, 30 eV, 40 eV. The data were acquired and analyzed with the Agilent Mass-Hunter Workstation Software (version B.07.00).

### 4.5. Quantitative Analysis of DH Herbal Pair

Quantitative analysis of DH herbal pair extract was performed on the Agilent 1290 liquid chromatography system. The conditions of the column and mobile phase in the HPLC analysis were applied for the separation of the compounds. A standard solution of danshensu, hydroxysafflor yellow A, and kaempferol-3-*O*-rutinoside was prepared at concentrations of 0.254 mg/mL, 0.444 mg/mL, and 0.125 mg/mL in 50% methanol and stored at 4 °C until use. A standard solution of rosmarinic acid, lithospermic acid, salvianolic acid B, and salvianolic acid A was prepared in concentrations of 0.192 mg/mL, 0.160 mg/mL, 0.425 mg/mL, and 0.156 mg/mL in methanol and stored at 4 °C until use. A standard solution of dihydrotanshinone I, cryptotanshinone, tanshinone I, and tanshinone IIA was dissolved completely in concentrations of 0.043 mg/mL, 0.103 mg/mL, 0.050 mg/mL, and 0.138 mg/mL in acetonitrile and stored at 4 °C until use. Standard working solution of mixtures of 11 analytes was obtained by diluting stock solutions to desired concentrations.

### 4.6. Method Validation

In order to validate the analytical performance of the established method, linearity, LODs and LOQs, precision, repeatability, stability, and recovery were carried out based on the International Conference on Harmonization (ICH) guideline. The linearity was determined by the ratio of corresponding peak areas and concentrations for each analyte. LODs and LOQs of the 11 compounds were acquired while the signal-to-noise ratios (S/N) were 3 and 10, respectively. Intra- and interday precisions were determined by analyzing the working standard solution continuously six times within a day and for three consecutive days. To confirm the repeatability, 6 independent samples were analyzed and the RSD% was obtained. For the stability testing, a single sample solution stored at room temperature was analyzed at 0, 2, 4, 6, 8, 18, and 24 h. A recovery test was conducted to confirm the accuracy of the developed analytical method. Comparable amounts of standards were added to the known contents of samples, and the combinational samples were analyzed with the developed procedure.

### 4.7. Zebrafish Husbandry and Model Construction

The AB strain zebrafish were bought from Nanjing YSY Biotech Co. Ltd. and maintained according to instructions in *The Zebrafish Handbook* [32]. They were raised separately under a 14:10 h light/dark cycle at 28 °C and fed twice daily with brine shrimp. To obtain zebrafish embryos, 3–4 pairs of zebrafish were placed in the spawning box for natural mating.

For the thrombosis model, the zebrafish embryos at the 2 dpf stage were exposed to 1.5 µM PHZ for 24 h [33]. After PHZ removal, zebrafish were cultured with DS, HH, and the DH herbal pair extracts in different proportions (50 µg/mL). The zebrafish dealt with 0.1% DMSO was used as the vehicle control, and aspirin (20 µg/mL) was a positive control. After 24 h, zebrafish from all groups were stained with 1.0 mg/mL *o*-dianisidine dye liquor for 15 min. The heart position of the zebrafish was observed and the image was captured with a stereomicroscope (Olympus Microsystems, Tokyo, Japan). The intensity of heart erythrocytes was quantitatively analyzed using Image-Pro Plus 6.0, and the anti-thrombotic efficacy was calculated with the following formula:

$${\mathrm{Efficacy}\;(\%)=[(\mathrm{SI}}_{\mathrm{drug}}{-\mathrm{SI}}_{\mathrm{model}}{)/(\mathrm{SI}}_{\mathrm{control}}{-\mathrm{SI}}_{\mathrm{model}})]\times 100\%$$

Both the BPF-induced zebrafish model and the ponatinib-induced ischemic stroke model were applied to explore the neuroprotection of the DH herbal pair. For the BPF-induced neurotoxicity model, zebrafish embryos aged 2 hpf were co-incubated with DS, HH, and eight DH herbal pair extracts (75 µg/mL) to 72 hpf in the presence of BPF (10 µg/mL). In the ponatinib-induced ischemic stroke model, 2 dpf zebrafish were treated with DS, HH, and eight DH herbal pairs (75 µg/mL) for 24 h in the presence of 1 μg/mL ponatinib. The zebrafish embryos treated with 0.1% DMSO were the vehicle control. After administration, the zebrafish were stained with 2.5 μg/mL acridine orange for 30 min in the dark. Subsequently, the staining solution was washed out and the zebrafish were fixed on glass slides. The fluorescence intensity (FI) in the brain was used to assess neuronal apoptosis, which displayed a yellow-green fluorescent signal under a stereomicroscope (Olympus Microsystems, Japan). The FI of the zebrafish brains was quantitatively analyzed using the Image J software, and the neuroprotection efficacy of drugs was calculated with the following formula:

$${\mathrm{Efficacy}\;(\%)=[(\mathrm{FI}}_{\mathrm{model}}{-\mathrm{FI}}_{\mathrm{drug}}{)/(\mathrm{FI}}_{\mathrm{model}}{-\mathrm{FI}}_{\mathrm{control}})]\times 100\%$$

### 4.8. Development of the Rat BCCAO Model and Drug Treatments

Adult male Wistar rats (220 ± 10 g) were purchased from Vital River Laboratories (Beijing, China) and randomly housed 3 per cage under a controlled temperature (24 ± 2 °C) for a 12:12-h light-dark cycle. Rats acclimated for 1 week with free access to standard food and water. All animal experiments were performed under the National Institutes of Health guide for the care and use of laboratory animals and approved by the Animal Ethics Committee of China Pharmaceutical University (No. 2021-10-007).

Rats were treated with BCCAO surgery to build the VaD model that was reported [34]. Rats were anesthetized with 3.5% isoflurane in an induction chamber (RWD Life Science, Shenzhen, China) for induction and then supplied with 1.5–2% isoflurane in a respiratory mask for maintenance during the surgery. In the BCCAO group, the bilateral common carotid arteries were exposed and ligated with 4-0 surgical sutures. The neck incision was sutured. Rats in the sham group were treated with the same operation except for ligation.

Seven days after surgery, all rats were subjected to the MWM test to screen for BCCAO rats meeting the criteria for cognitive dysfunction. Animals were divided into five groups: (1) sham group; (2) model group; (3) BCCAO + DH herbal pair extract (3.2 g/kg/d, i.g.); (4) BCCAO + MCC (according to their content in equivalent DH herbal pair, i.g.); and (5) BCCAO + nicergoline (5 mg/kg, i.g.). Each group contained 11 rats. After 28 days of drug administration, the MWM test was conducted to evaluate the learning and memory functions of the animals.

After behavioral evaluation, the rats were euthanized, and their brains were dissected. Brains from each group were perfused with normal saline and paraformaldehyde to remove residual blood, and then they were fixed with 4% paraformaldehyde and embedded with paraffin for staining experiments. For the measurement of ACh content and AChE activity, the hippocampi of rats were quickly stripped away for detection. The detailed experimental protocol is shown in Figure 6A.

### 4.9. Morris Water Maze Test (MWM)

For evaluating cognitive impairment, the MWM test was performed as previously described [35]. The first stage was carried out 7 days after BCCAO surgery to evaluate if the rat VaD model was successfully established, and the second was performed after drug administration to evaluate the learning and memory dysfunction of animals and the therapeutic effects of drugs. Specifically, the MWM consisted of a circular pool of 2.0 m in diameter with water (25 ± 1 °C) and a submerged escape platform placed in the center of the first quadrant one inch above the water. For pretraining (three trials per day for 5 d), rats were released into the water facing the sidewalls from different quadrants and allowed 120 s to locate the platform. Swimming speed (cm/s) and escape latency (s) were recorded. If animals did not find the platform within 120 s, they were guided to board the platform and allowed to stay on the platform for 10 s. On day 6, the platform was removed. The quadrant farthest from the original platform was selected as the entry point for the animals. The platform crossing time and the target quadrant time percentage (%) within 120 s were recorded.

### 4.10. Measurement of ACh Content and AChE Activity

The hippocampal tissues were homogenized with normal saline or a specified reagent. After centrifugation, the supernatant of the homogenate was collected. The Ach content and AChE activity in the hippocampus were determined according to the steps of the acetylcholine assay kit (Nanjing Jiancheng, A105-1-1) and the acetylcholinesterase assay kit (Nanjing Jiancheng, A024-1-1), respectively.

### 4.11. Nissl Staining and Dihydroethidium (DHE) Staining

The brain tissues were immobilized in 4% paraformaldehyde and embedded in paraffin; then, 20 µm-thick sections were prepared. For Nissl staining, sections containing hippocampal tissues were stained with cresyl violet (Nissl) stain solution, dehydrated through graded ethanol, and then cleared with xylene. The morphology of neurons in the hippocampus was observed and the number of Nissl staining-positive neurons was counted under a light microscope (Nikon, DS-Ri2, Tokyo, Japan). For DHE staining, slices were co-incubated with a DHE reagent at 37 °C for 30 min, and the fluorescence intensity of ROS in the hippocampus was detected under a confocal laser-scanning microscope (Zeiss, LSM 800, Jena, Germany).

### 4.12. The Spectrum-Effect Relationship Analysis

The spectrum-effect relationship analysis was performed with the SIMCA14.0 software, connecting the efficacy of different DH herbal pair extracts on three zebrafish models with the peak area of the identified compounds in the corresponding samples. The correlation was calculated using the PLS model, and the compounds with a value of VIP > 1 were regarded as closely related to the pharmacological effects.

### 4.13. Statistical Analysis

All data are presented as mean ± SEM. The statistical analyses were performed using GraphPad Prism 8.0, and one-way ANOVA was utilized to compare the between-group differences, followed by Tukey’s test. All experiments were randomized and blinded and were repeated at least three times to confirm their reproducibility. Statistical significance was defined as *p* < 0.05.

## Figures and Tables

**Table 1:** Component identification of the Danshen-Honghua (DH) herbal pair.

No.	T_R_ (min)	Ion Mode	*m/z*	Calculate (*m/z*)	Error (ppm)	Fragment Ions (*m/z*)	Formula	Identification
1	2.04	[M−H]^−^	128.0363	128.0353	−5.30	128.04, 82.03	C_5_H_7_NO_3_	L-Pyroglutamic acid
2	2.28	[M+H]^+^	268.1035	268.1040	1.99	136.06, 119.03	C_10_H_13_N_5_O_4_	Adenosine
3	2.73	[M−H]^−^	117.0196	117.0193	−2.27	117.02, 99.01, 73.03	C_4_H_6_O_4_	Succinic acid
4	3.53	[M−H]^−^	299.0766	299.0772	2.14	137.03, 93.04	C_13_H_16_O_8_	4-Hydroxybenzoic acid 4-*O*-glucoside
5	3.96	[M+H]^+^	166.0860	166.0863	1.55	120.08, 103.05, 91.06, 77.04	C_9_H_11_NO_2_	Phenylalanine
6	4.49	[M+H]^+^	127.0388	127.0390	1.35	109.03, 81.03, 53.04	C_6_H_6_O_3_	5-Hydroxymethylfurfural
7	4.80	[M−H]^−^	611.1604	611.1618	2.22	491.12, 473.11, 403.10, 325.08, 283.06, 163.01, 119.05	C_27_H_32_O_16_	Hydroxysafflor yellow A isomer
8	5.02	[M−H]^−^	197.0452	197.0455	1.75	179.04, 135.05, 109.03	C_9_H_10_O_5_	Danshensu ^a^
9	5.91	[M−H]^−^	153.0198	153.0193	−3.04	109.03, 91.02, 53.05	C_7_H_6_O_4_	Protocatechuic acid ^a^
10	6.86	[M−H]^−^	353.0878	353.0878	0.02	191.05, 179.03, 173.04, 161.03, 135.04	C_16_H_18_O_9_	Neochlorogenic acid ^a^
11	7.14	[M+H]^+^	205.0965	205.0972	3.20	188.07, 146.06, 118.06	C_11_H_12_N_2_O_2_	L-Tryptophan
12	7.62	[M−H]^−^	181.0506	181.0506	0.18	163.04, 135.04, 117.03, 92.92, 53.75	C_9_H_10_O_4_	3,4-Dihydroxy benzenepropionic acid
13	8.85	[M−H]^−^	137.0239	137.0244	3.75	119.01, 109.03, 108.02, 91.02	C_7_H_6_O_3_	Protocatechualdehyde ^a^
14	9.37	[M−H]^−^	137.0243	137.0244	1.58	125.35, 94.04, 93.03, 88.20, 81.53	C_7_H_6_O_3_	*p*-Hydroxybenzoic acid ^a^
15	9.91	[M−H]^−^	153.0186	153.0193	4.75	109.03, 79.02	C_7_H_6_O_4_	3,5-Dihydroxybenzonic acid/isomer
16	10.13	[M−H]^−^	341.0876	341.0878	0.60	179.04, 135.04	C_15_H_18_O_9_	Caffeic acid-*O*-hexoside
17	10.52	[M−H]^−^	787.1937	787.1938	0.19	625.14, 463.09, 301.03	C_33_H_40_O_22_	6-Hydroxykaempferol-3,6,7-triglucoside
18	10.72	[M−H]^−^	353.0882	353.0878	−1.33	353.09, 191.05, 85.03	C_16_H_18_O_9_	Chlorogenic acid ^a^
19	11.33	[M−H]^−^	611.1625	611.1618	−1.21	611.16, 491.12, 473.11, 403.10, 325.07, 313.07, 295.06, 283.06, 207.05, 205.01, 163.01, 119.05	C_27_H_32_O_16_	Hydroxysafflor yellow A ^a^
20	11.46	[M−H]^−^	325.0938	325.0929	−2.79	163.04, 119.05	C_15_H_18_O_8_	Coumaric acid-*O*-hexoside
21	12.36	[M−H]^−^	353.0876	353.0878	0.58	191.05, 179.03, 173.04, 135.04	C_16_H_18_O_9_	Cryptochlorogenic acid ^a^
22	12.96	[M−H]^−^	179.0347	179.0350	1.57	179.03, 135.04, 89.04	C_9_H_8_O_4_	Caffeic acid ^a^
23	13.32	[M+H]^+^	627.1540	627.1556	2.52	465.10, 303.05	C_27_H_30_O_17_	6-Hydroxykaempferol-di-*O*-glucoside
24	14.77	[M−H]^−^	595.1664	595.1668	0.74	505.14, 385.09, 313.07	C_27_H_32_O_15_	Eriocitrin
25	14.82	[M+H]^+^	627.1566	627.1556	0.12	465.10, 303.05	C_27_H_30_O_17_	6-Hydrokaempferol-di-*O*-glucoside
26	16.09	[M+COOH]^−^	431.1925	431.1923	−0.53	385.19, 223.13, 205.12, 153.09	C_19_H_30_O_8_	Roseoside
27	16.54	[M−H]^−^	357.1189	357.1191	0.57	195.07, 119.05	C_16_H_22_O	Sweroside
28	19.48	[M−H]^−^	503.1779	503.1770	−1.76	355.09, 337.08, 193.04, 89.02	C_22_H_32_O_13_	Tinosinen
29	20.01	[M−H]^−^	163.0405	163.0401	−2.64	119.05, 93.04, 63.90	C_9_H_8_O_3_	*p*-coumaric acid ^a^
30	20.39	[M−H]^−^	611.1635	611.1618	−2.84	521.123, 491.11, 448.10, 313.07, 207.05, 119.05	C_27_H_32_O_16_	Hydroxysafflor yellow B
31	21.19	[M−H]^−^	325.0937	325.0929	−2.48	163.04, 119.05	C_15_H_18_O_8_	(2Z)-2-(Glucopyranosyloxy)-3-phenyl-2-propenoic acid
32	21.54	[M−H]^−^	771.2003	771.1989	−1.77	609.15, 301.04	C_33_H_40_O_21_	6-Hydroxykaempferol-3-*O*-rutinoside-6-*O*-glucoside
33	21.70	[M−H]^−^	625.1424	625.1410	−2.20	463.09, 301.03	C_27_H_30_O_17_	6-Hydroxykaempferol-3,6-di-*O*-glucoside
34	25.24	[M+H]^+^	611.1603	611.1607	0.59	449.10, 287.05	C_27_H_30_O_16_	kaempferol 3-*O*-sophoroside ^a^
35	26.99	[M−H]^−^	609.1472	609.1461	−1.79	301.04, 255.03	C_27_H_30_O_16_	Rutin ^a^
36	27.51	[M−H]^−^	463.0886	463.0882	−0.86	301.04, 185.87	C_21_H_20_O_12_	Quercetin-7-*O*-glucoside ^a^
37	29.54	[M−H]^−^	449.1102	449.1089	−2.81	329.06, 287.06	C_21_H_22_O_11_	Neocarthamin
38	31.89	[M−H]^−^	521.1324	521.1301	−4.47	359.08, 323.08, 197.04, 179.04, 161.03	C_24_H_26_O_13_	Salviaflaside
39	32.54	[M−H]^−^	593.1529	593.1512	−2.87	473.09, 285.04, 255.03, 227.03	C_27_H_30_O_15_	Kaempferol 3-*O*-rutinoside ^a^
40	32.87	[M−H]^−^	1043.2690	1043.2674	−1.53	1025.26, 923.23, 863.20, 593.15, 449.11, 407.10, 287.06	C_48_H_52_O_26_	Anhydrosafflor yellow B ^a^
41	33.71	[M−H]^−^	537.1047	537.1038	−1.58	493.09, 339.05, 321.04, 313.07, 295.06, 277.05, 269.09, 267.07, 253.05, 185.02, 179.04, 135.05	C_27_H_22_O_12_	Salvianolic acid H
42	33.92	[M−H]^−^	623.1627	623.1618	−1.51	315.05, 271.02	C_28_H_32_O_16_	Isorhamnetin-3-*O*-rutinoside
43	34.43	[M−H]^−^	447.0933	447.0933	−0.03	284.03, 151.00	C_21_H_20_O_11_	Luteoloside
44	36.03	[M−H]^−^	477.1034	477.1038	0.94	462.08, 315.05, 300.03, 271.03, 209.01, 170.05, 106.04	C_22_H_22_O_12_	Safloroside
45	40.26	[M−H]^−^	537.1018	537.1038	3.81	339.05, 321.05, 295.06, 277.06, 185.01, 159.04	C_27_H_22_O_12_	Salvianolic acid I
46	41.11	[M−H]^−^	359.0773	359.0722	−0.16	197.04, 179.03, 161.02, 135.04, 133.03, 123.04	C_18_H_16_O_8_	Rosmarinic acid ^a^
47	41.80	[M−H]^−^	717.1456	717.1461	0.71	537.11, 519.09, 493.12, 339.05, 321.04, 295.06, 279.03, 185.03	C_36_H_30_O_16_	Salvianolic acid E
48	45.04	[M−H]^−^	537.1037	537.1038	0.28	493.11, 313.07, 295.06, 277.06, 269.07, 267.09, 203.04, 197.04, 185.02, 135.04, 109.03	C_27_H_22_O_12_	Lithospermic acid ^a^
49	46.23	[M−H]^−^	613.1561	613.1563	0.29	551.16, 361.11, 287.06	C_30_H_30_O_14_	Safflomin C
50	48.15	[M−H]^−^	613.1570	613.1563	−1.17	551.15, 361.11, 287.06	C_30_H_30_O_14_	Safflomin C isomer
51	49.39	[M−H]^−^	717.1470	717.1461	−1.24	717.14, 673.16, 537.10, 519.09, 493.11, 475.10, 457.09, 377.09, 339.05, 321.04, 295.06, 293.05, 197.05, 185.02, 135.05, 109.03	C_36_H_30_O_16_	Salvianolic acid B ^a^
52	50.40	[M−H]^−^	717.1469	717.1461	−1.10	519.09, 339.05, 321.04, 295.06, 185.02, 135.04	C_36_H_30_O_16_	Salvianolic acid L
53	51.70	[M−H]^−^	373.0923	373.0929	1.58	179.03, 135.05	C_19_H_18_O_8_	Rosmarinic acid methyl ester
54	52.63	[M+H]^+^	311.1481	311.1489	2.63	149.09, 127.07, 85.03	C_16_H_22_O_16_	(2Z)-2-decene-4,6-diyn-1-yl-Glucopyranoside
55	53.38	[M−H]^−^	493.1155	493.1140	−2.99	383.10, 313.07, 295.06, 277.05, 267.07, 249.06, 203.04, 197.05, 185.03, 159.05, 135.05, 109.03	C_26_H_22_O_10_	Salvianolic acid A ^a^
56	55.17	[M−H]^−^	731.1623	731.1618	−0.74	533.11, 515.10, 507.1310, 353.07, 335.06, 327.08, 320.04, 309.07, 197.05, 179.03, 135.04	C_37_H_32_O_16_	3′-methyl Salvianolic acid B
57	59.79	[M−H]^−^	565.1338	565.1351	2.38	385.09, 367.08, 321.04, 293.05, 277.05, 257.05, 245.05, 231.07, 179.04	C_29_H_26_O_12_	Dimethyl lithospermic acid
58	62.75	[M−H]^−^	491.0991	491.0984	−1.48	311.0556, 293.0447, 267.07, 265.06, 197.04, 135.04	C_26_H_20_O_10_	Salvianolic acid C ^a^
59	72.96	[M+H]^+^	311.1268	311.1278	3.18	293.12, 278.09, 275.11, 265.10, 251.10, 247.11, 232.08, 219.11, 204.09	C_19_H_18_O_4_	Tanshinone IIB
60	75.73	[M+H]^+^	341.1377	341.1384	1.91	341.1391, 281.1161, 263.11, 235.11, 192.08	C_20_H_20_O_5_	Trijuganone C
61	77.24	[M+H]^+^	293.0797	293.0808	3.89	293.08, 275.76, 266.09, 265.08, 250.06, 247.07, 237.08, 222.07, 219.08, 209.09, 194.08, 191.08	C_18_H_12_O_4_	Monohydroxytanshinone I
62	78.28	[M+H]^+^	281.1536	281.1536	0.02	266.12, 263.14, 253.13, 252.12, 239.11, 238.10, 235.16, 222.26, 211.10, 208.09, 197.13	C_19_H_20_O_2_	Sibiriquinone A
63	78.46	[M+H]^+^	293.1174	293.1172	−0.61	278.09, 275.10, 263.07, 251.07, 247.11, 235.07, 232.08, 229.11, 219.12, 204.09, 193.10, 189.07, 179.09, 167.08	C_19_H_16_O_3_	1,2-Didehydrotanshinone IIA
64	79.74	[M+H]^+^	279.1011	279.1016	1.69	261.09, 251.09, 246.07, 233.10, 223.08, 218.07, 205.10, 190.08, 169.06, 141.07	C_18_H_14_O_3_	Dihydrotanshinone I ^a^
65	81.26	[M+H]^+^	281.1179	281.1172	−2.42	281.12, 263.11, 248.08, 235.11, 220.09, 217.10, 207.11, 202.07, 192.09, 179.09, 169.06, 165.07	C_18_H_16_O_3_	Trijuganone B
66	82.31	[M+H]^+^	339.1213	339.1217	4.14	311.13, 279.10, 261.09, 233.10, 190.08	C_20_H_18_O_5_	Methyl tanshinonate
67	85.24	[M+H]^+^	297.1479	297.1485	2.10	282.13, 279.14, 268.11, 264.11, 254.09, 251.14, 237.10, 236.11, 233.13, 223.14, 208.11, 197.09, 195.08, 193.10, 181.10	C_19_H_20_O_3_	Cryptotanshinone ^a^
68	86.08	[M+H]^+^	277.0860	277.0859	0.29	259.07, 249.09, 234.07, 231.08, 221.10, 206.07, 203.08, 193.10, 178.08	C_18_H_12_O_3_	Tanshinone I ^a^
69	87.13	[M+H]^+^	265.1215	265.1223	3.05	265.12, 247.11, 232.09, 223.08, 219.12, 204.09, 194.11, 179.08, 167.09	C_18_H_16_O_2_	R0-090680
70	89.40	[M+H]^+^	281.1534	281.1536	0.47	266.13, 263.14, 253.16, 248.11, 239.13, 238.12, 235.15, 233.09, 225.09, 211.13, 221.09, 193.10, 149.02	C_19_H_20_O_2_	1,2-Didehydromiltirone
71	90.20	[M+H]^+^	295.1321	295.1329	2.62	280.11, 277.12, 262.10, 253.08, 252.08, 249.13, 235.08, 234.10, 231.12, 221.13, 207.09, 206.11, 191.08	C_19_H_18_O_3_	Tanshinone IIA ^a^
72	91.14	[M+H]^+^	283.1689	283.1693	1.26	268.15, 265.16, 250.13, 241.12, 240.11, 237.16, 225.10, 223.11, 208.09, 195.11, 180.10, 167.08	C_19_H_22_O_2_	Miltirone

^a^ Further confirmed with reference peaks.

**Table 2:** Antithrombotic and neuroprotective efficacy of the DH herbal pair.

Sample	Antithrombotic Rate (PHZ)	Neuroprotection Rate (BPF)	Neuroprotection Rate (Ponatinib)
DS	36.36% ^a,**^	21.54% ^b,ns^	64.60% ^b,****^
DH 5:1	38.49% ^***^	26.75% ^*^	66.14% ^****^
DH 4:1	34.38% ^**^	27.33% ^*^	71.06% ^****^
DH 3:1	41.20% ^***^	31.65% ^**^	74.99% ^****^
DH 2:1	34.09% ^**^	25.34% ^ns^	70.01% ^****^
DH 1:1	39.04% ^***^	31.57% ^**^	63.45% ^****^
DH 1:2	31.92% ^*^	27.09% ^*^	69.44% ^****^
HH	36.97% ^**^	20.17% ^ns^	67.16% ^****^

^a^ ${\mathrm{Antithrombotic}\;\mathrm{rate}\;(\%)=[(\mathrm{SI}}_{\mathrm{drug}}{-\mathrm{SI}}_{\mathrm{model}}{)/(\mathrm{SI}}_{\mathrm{control}}{-\mathrm{SI}}_{\mathrm{model}})]\times 100\%$, SI: staining intensity of erythrocytes in the heart; ^b^ ${\mathrm{neuroprotection}\;\mathrm{rate}\;(\%)=[(\mathrm{FI}}_{\mathrm{model}}{-\mathrm{FI}}_{\mathrm{drug}}{)/(\mathrm{FI}}_{\mathrm{model}}{-\mathrm{FI}}_{\mathrm{ctrl}})]\times 100\%$, FI: fluorescence intensity of zebrafish brain; DS: Danshen; HH: Honghua; DH: Danshen-Honghua; * *p* < 0.05, ** *p* < 0.01, *** *p* < 0.001, **** *p* < 0.0001 vs. model group, ns: no significant difference.

**Table 3:** Summary of the VIP values of the components in the DH herbal pair in three zebrafish models.

Model	Peak Number														
	**1**	**2**	**3**	**4**	**5**	**6**	**7**	**8**	**9**	**10**	**11**	**12**	**13**	**14**	**15**
**PHZ**	0.7521	0.5154	0.5134	0.2658	0.5549	0.1648	0.4273	1.1379 *	0.1276	0.4321	0.5204	0.1416	0.2747	0.1825	0.1582
**BPF**	0.7342	0.4950	0.4993	0.2660	0.5378	0.2254	0.4101	1.1467 *	0.1744	0.4127	0.5037	0.1939	0.3141	0.1898	0.1578
**Ponatinib**	0.7621	0.5218	0.5166	0.2721	0.5558	0.1571	0.4324	1.1388 *	0.1217	0.4414	0.5217	0.1351	0.2739	0.1948	0.1748
	**16**	**17**	**18**	**19**	**20**	**21**	**22**	**23**	**24**	**25**	**26**	**27**	**28**	**29**	**30**
**PHZ**	0.6944	0.5726	0.8449	1.7532 *	0.2560	0.2815	0.5518	0.4626	0.5997	0.3404	0.2235	0.3138	0.2133	0.8976	0.3111
**BPF**	0.6975	0.5483	0.8080	1.6917 *	0.2505	0.2788	0.5500	0.4359	0.5930	0.3138	0.2394	0.3115	0.2913	0.9546	0.4250
**Ponatinib**	0.7035	0.5802	0.8547	1.7750 *	0.2610	0.2860	0.5511	0.4675	0.6078	0.3391	0.2351	0.3208	0.2034	0.9323	0.2967
	**31**	**32**	**33**	**34**	**35**	**36**	**37**	**38**	**39**	**40**	**41**	**42**	**43**	**44**	**45**
**PHZ**	0.5635	0.5113	0.8562	0.7700	0.3947	0.3917	0.5786	0.4836	1.0373 *	0.7676	0.6828	0.6586	0.3385	0.4838	0.2182
**BPF**	0.5463	0.5035	0.8407	0.7447	0.3976	0.3769	0.5505	0.4848	1.0025 *	0.7343	0.6842	0.6492	0.3354	0.4696	0.2980
**Ponatinib**	0.5686	0.5140	0.8634	0.7773	0.4000	0.3983	0.5819	0.4819	1.0441 *	0.7737	0.6773	0.6722	0.3451	0.4898	0.2081
	**46**	**47**	**48**	**49**	**50**	**51**	**52**	**53**	**54**	**55**	**56**	**57**	**58**	**59**	**60**
**PHZ**	1.6341 *	0.6784	1.4893 *	0.3097	0.3815	5.9960 *	0.7086	0.5442	0.6152	1.5265 *	0.3003	0.3915	0.8039	0.5504	0.3459
**BPF**	1.6390 *	0.6806	1.4975 *	0.2921	0.3698	6.0138 *	0.7099	0.5456	0.5841	1.5573 *	0.3023	0.3918	0.8083	0.5500	0.3528
**Ponatinib**	1.6305 *	0.6761	1.4866 *	0.3115	0.3863	5.9798 *	0.7083	0.5436	0.6222	1.5242 *	0.2972	0.3884	0.8010	0.5460	0.3411
	**61**	**62**	**63**	**64**	**65**	**66**	**67**	**68**	**69**	**70**	**71**	**72**			
**PHZ**	0.3040	0.3391	0.5084	1.0073 *	0.6035	0.4975	1.3229 *	1.2337 *	0.4814	0.5069	1.8227 *	0.5298			
**PDF**	0.3217	0.3392	0.5170	1.0088 *	0.6094	0.4992	1.3258 *	1.2423 *	0.4627	0.5218	1.8252 *	0.5309			
**Ponatinib**	0.3038	0.3354	0.5062	1.0017 *	0.5979	0.4944	1.3152 *	1.2265 *	0.4812	0.5166	1.8115 *	0.5269			

* means VIP > 1.

**Table 4:** Content of 11 compounds in the multi-component combination (MCC).

Peaks	Content (mg/g)	RSD (%)
Danshensu	3.03 ± 0.05	1.77
Hydroxysafflor yellow A	15.85 ± 0.19	1.19
Kaempferol-3-*O*-rutinoside	0.89 ± 0.01	1.64
Rosmarinic acid	2.15 ± 0.02	1.11
Lithospermic acid	2.83 ± 0.04	1.51
Salvianolic acid B	17.31 ± 0.20	1.20
Salvianolic acid A	1.82 ± 0.02	1.25
Dihydroisotanshinone I	0.54 ± 0.01	2.20
Cryptotanshinone	2.76 ± 0.08	3.09
Tanshinone I	0.93 ± 0.02	2.59
Tanshinone ⅡA	2.09 ± 0.07	3.32

RSD: relative standard deviation; n = 6.

## Data Availability

The data presented in this study are available within the article and supplementary materials.

## Supplementary Materials

The following supporting information can be downloaded at: https://www.mdpi.com/article/10.3390/ph15091073/s1, Figure S1: The total ion chromatograms (TIC) of Danshen-Honghua (DH) herbal pair extracted by ultra-high-performance liquid chromatography coupled with quadrupole time-of-flight tandem mass spectrometry (UHPLC-QTOF MS) in positive- and negative-ion mode. Table S1: Death number of zebrafish in different concentrations of eight DH herbal pair in phenylhydrazine (PHZ)-induced thrombosis model. Table S2: Death number of zebrafish in different concentrations of DH herbal pair in bisphenol F (BPF)-induced neuronal injury model. Table S3: Death number of zebrafish in different concentrations of eight DH herbal pair in a ponatinib-induced ischemic stroke model. Table S4: Standard curves, limits of detection, limits of quantification, precision (intra-day, inter-day), repeatability, stability, and accuracy for the 11 components measured.

## Abbreviations

The following abbreviations are used in this manuscript.

VaD	Vascular dementia
AD	Alzheimer’s disease
ROS	Reactive oxygen species
TCMCR	Traditional Chinese medicine compound recipe
DS	Danshen
HH	Honghua
DH	Danshen-Honghua
DHI	Danhong injection
BCCAO	Bilateral common carotid artery occlusion
UHPLC-QTOF MS	Ultra-high-performance liquid chromatography-quadrupole time-of-flight mass spectrometry
TIC	Total ion chromatograms
MS	Mass Spectrometry
PHZ	Phenylhydrazine
MNLC	Maximum nonlethal concentration
RBCs	Red blood cells
BPF	Bisphenol F
PLS	Partial least squares
HPLC	High performance liquid chromatography
VIP	Variable importance in projection
MCC	Multi-component combination
r^2^	Correlation coefficients
LODs	Limits of detection
LOQs	Limits of quantification
RSD	Relative standard deviations
MWM	Morris water maze
ACh	Acetylcholine
AChE	Acetylcholinesterase
DHE	Dihydroethidium
TCM	Traditional Chinese medicine
BECCs	Bioactive equivalent combinatorial components
DMSO	Dimethyl sulfoxide
FI	Fluorescence intensity
SI	Staining intensity
ESI	Electrospray ionization
ICH	International Conference on Harmonization
S/NCNS	Signal-to-noise ratiosCentral nervous system
