# Supplementary material for: Discovering a Multi-Component Combination against Vascular Dementia from Danshen-Honghua Herbal Pair by Spectrum-Effect Relationship Analysis
Source: Pharmaceuticals (Basel). 2022 Aug 29;15(9):1073. doi: 10.3390/ph15091073 (PMC9505896; doi:10.3390/ph15091073)
Supplement: Supplementary file 1 [file pharmaceuticals-15-01073-s001.zip › pharmaceuticals-1860970-supplementary.pdf]

Supplementary Materials

Discovering a multi-component combination against vascular dementia from Danshen-Honghua herbal pair by spectrum-effect relationship analysis

Peilin Zhang[†], Shiru He[†], Siqi Wu, Yi Li, Huiying Wang, Changyang Yan, Hua Yang*, Ping Li*

State Key Laboratory of Natural Medicines, China Pharmaceutical University, No. 24 Tongjia Lane, Nanjing 210009, China

[†] These authors contributed equally to this work.

* Corresponding authors: China Pharmaceutical University, No. 24, Tongjia Lane, Nanjing, China. Tel: +86 25 8327 1379; fax: +86 25 8327 1379.

E-mail addresses: 104yang104@163.com (H. Yang), liping2004@126.com (P. Li).

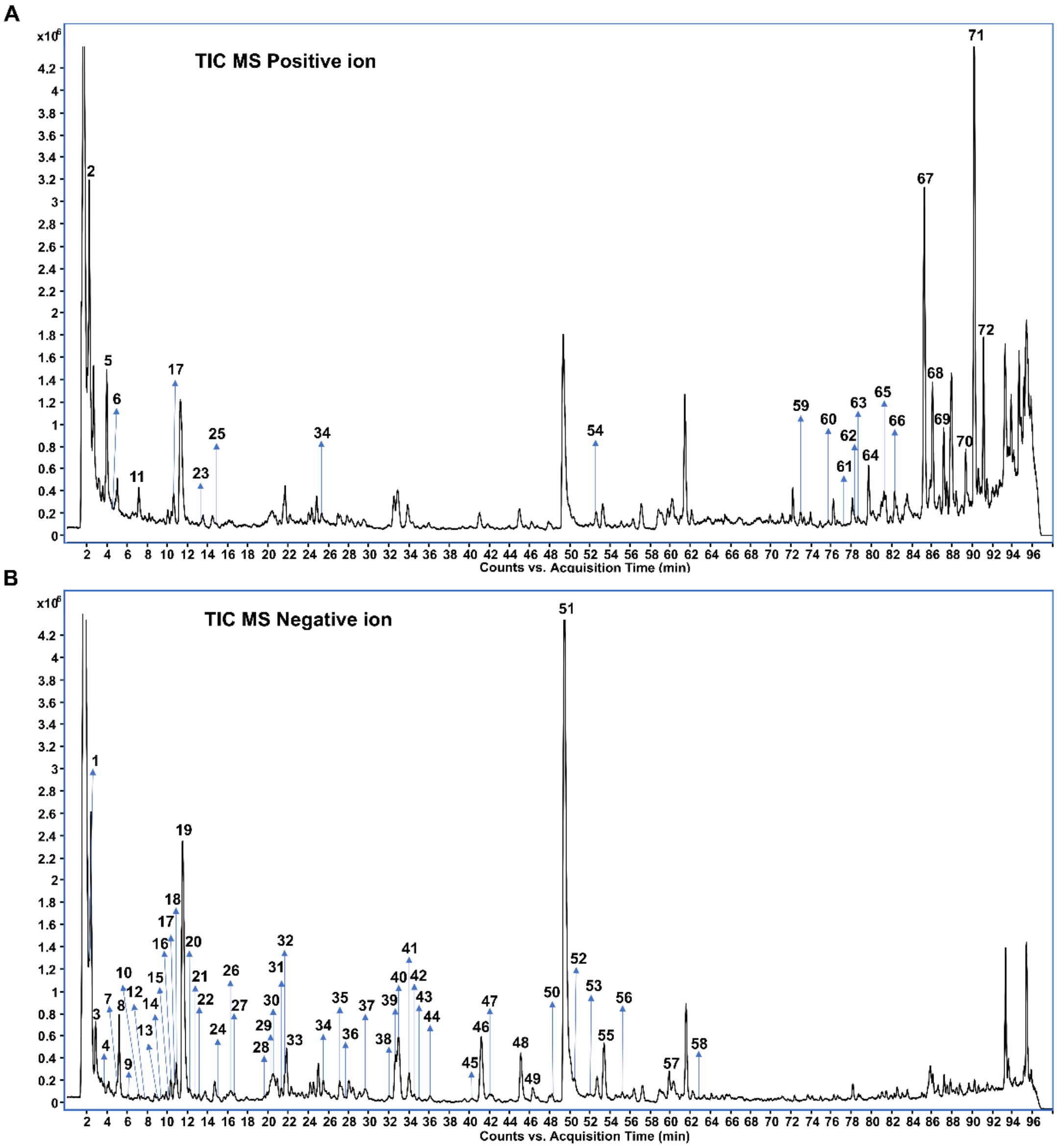

Figure S1. The total ion chromatograms (TIC) of Danshen-Honghua (DH) herbal pair extracted by ultra-high performance liquid chromatography coupled with quadrupole time-of-flight tandem mass spectrometry (UHPLC-QTOF MS) in （A） positive- and (**B**) negative-ion mode.

Table S1. Death number of zebrafish in different concentrations of eight DH herbal pair in phenylhydrazine (PHZ)-induced thrombosis model.

	**600 μg/mL**	**300 μg/mL**	**150 μg/mL**	**75 μg/mL**	**50 μg/mL**
DS	30	30	30	3	0
DH 5:1	30	30	21	0	0
DH 4:1	30	30	30	2	0
DH 3:1	30	30	22	0	0
DH 2:1	30	30	17	0	0
DH 1:1	30	24	15	0	0
DH 1:2	30	22	11	0	0
HH	30	20	13	0	0

DS: Danshen; HH: Honghua; DH: Danshen-Honghua; n = 30.

Table S2. Death number of zebrafish in different concentrations of eight DH herbal pair in bisphenol F (BPF)-induced neuronal injury model.

	**600 μg/mL**	**300 μg/mL**	**150 μg/mL**	**75 μg/mL**	**50 μg/mL**
DS	30	30	17	0	0
DH 5:1	30	30	13	0	0
DH 4:1	30	30	18	0	0
DH 3:1	30	25	16	0	0
DH 2:1	30	15	3	0	0
DH 1:1	26	10	3	0	0
DH 1:2	24	10	2	0	0
HH	22	9	1	0	0

DS: Danshen; HH: Honghua; DH: Danshen-Honghua; n = 30.

Table S3. Death number of zebrafish in different concentrations of eight DH herbal pair in ponatinib-induced ischemic stroke model.

	**600 μg/mL**	**300 μg/mL**	**150 μg/mL**	**75 μg/mL**	**50 μg/mL**
DS	30	30	24	0	0
DH 5:1	30	29	0	0	0
DH 4:1	30	30	6	0	0
DH 3:1	30	30	21	0	0
DH 2:1	30	20	1	0	0
DH 1:1	10	1	0	0	0
DH 1:2	10	3	0	0	0
HH	8	6	0	0	0

DS: Danshen; HH: Honghua; DH: Danshen-Honghua; n = 30.

Table S4. Standard curves, limits of detection, limits of quantification, precision (intra-day, inter-day), repeatability, stability, and accuracy for 11 components measured.

Analytes	Regression equation	r^2	Test range (μg/mL)	LODs (μg/mL)	LOQs (μg/mL)	Precision (RSD%)		Repeatability (RSD%)	Stability (RSD%)	Recovery rate%
						Inter-day	Intra-day			
Danshensu	y = 3.2869x – 8.1043	0.9997	3.97-254.54	0.020	0.199	0.20	2.07	1.77	1.41	101.80 ± 3.12
Hydroxysafflor yellow A	y = 7.2194x – 38.216	0.9998	10.48-671.10	0.010	0.052	0.35	2.81	1.19	1.11	100.0 4 ± 3.27
Kaempferol-3-*O*-rutinoside	y = 3.8949x – 5.4964	0.9996	1.95-125.00	0.195	0.977	0.73	0.50	1.64	1.25	105.47 ± 4.04
Rosmarinic acid	y = 11.646x – 18.487	0.9998	3.00-192.00	0.015	0.150	0.31	1.99	1.11	0.97	98.98 ± 4.03
Lithospermic acid	y = 6.0798x – 6.6936	0.9998	2.50-160.00	0.003	0.250	0.20	2.62	1.51	1.01	97.18 ± 2.42
Salvianolic acid B	y = 15.236x – 43.857	0.9999	6.64-425.00	0.003	0.007	0.36	1.96	1.20	0.93	100.01 ± 3.19
Salvianolic acid A	y = 12.192x – 26.213	0.9994	2.44-156.25	0.041	0.244	1.69	1.62	1.25	0.97	101.04 ± 2.12
Dihydroisotanshinone I	y = 16.926x – 6.9338	0.9997	0.68-43.68	0.022	0.087	0.75	1.64	2.2	0.47	103.14 ± 3.38
Cryptotanshinone	y = 6.799x – 4.9258	0.9998	1.61-103.12	0.052	0.206	0.79	1.92	3.09	0.58	98.89 ± 1.13
Tanshinone I	y = 14.407x – 2.3377	1	0.78-50.00	0.010	0.025	0.71	1.51	2.59	1.03	97.27 ± 2.93
Tanshinone IIA	y = 15.416x +2.2662	0.9999	2.16-138.46	0.000	0.014	0.75	2.24	3.32	0.63	95.76 ± 2.13

r^2: correlation coefficients; LODs: limits of detection; LOQs: limits of quantification; RSD: relative standard deviations.
